# The interplay of LncRNA ANRIL and miR‐181b on the inflammation‐relevant coronary artery disease through mediating NF‐κB signalling pathway

**DOI:** 10.1111/jcmm.13790

**Published:** 2018-08-05

**Authors:** Feng Guo, Chengchun Tang, Yawei Li, Yuqing Liu, Ping Lv, Wei Wang, Yongyong Mu

**Affiliations:** ^1^ Department of Emergency The 455th Hospital of Chinese People's Liberation Army Shanghai China; ^2^ Department of Cardiology Zhongda Hospital Southeast University Nanjing China

**Keywords:** coronary artery disease, lncRNA ANRIL, mice model, miR‐181b, NF‐κB

## Abstract

This study was designed to investigate whether ANRIL affected the aetiology of coronary artery disease (CAD) by acting on downstream miR‐181b and NF‐κB signalling. Altogether 327 CAD patients diagnosed by angiography were included, and mice models of CAD were established. Human coronary endothelial cells (HCAECs) and human umbilical vein endothelial cells (HUVECs) were also purchased. In addition, shRNA‐ANRIL, shRNA‐NC, pcDNA3.1‐ANRIL, miR‐181b mimic, miR‐181b inhibitor and miR‐NC were transfected into the cells. The lipopolysaccharides (LPS) and pyrrolidine dithiocarbamate (PDTC) were also added to activate or deactivate NF‐κB signalling. Both highly expressed ANRIL and lowly expressed miR‐181b were associated with CAD population aged over 60 years old, with smoking history, with hypertension and hyperlipidemia, with CHOL H 4.34 mmol/L, TG ≥ 1.93 mmol/L and Hcy ≥ 16.8 μmol/L (all *P* < 0.05). Besides, IL‐6, IL‐8, NF‐κB, TNF‐α, iNOS, ICAM‐1, VCAM‐1 and COX‐2 expressions observed within AD mice models were all beyond those within NC and sham‐operated groups (*P* < 0.05). Also VEGF and HSP 70 were highly expressed within AD mice models than within NC and sham‐operated mice (*P* < 0.05). Transfection of either pcDNA‐ANRIL or miR‐181b inhibitor could significantly fortify HCAECs’ viability and put on their survival rate. At the meantime, the inflammatory factors and vascular‐protective parameters were released to a greater level (*P* < 0.05). Finally, highly expressed ANRIL also notably bring down miR‐181b expression and raise p50/p65 expressions within HCAECs (*P* < 0.05). The joint role of ANRIL, miR‐181b and NF‐κB signalling could aid in further treating and diagnosing CAD.

## 1 INTRODUCTION

The cardiovascular and cerebrovascular disorders, especially coronary artery disease (CAD), have cast huge burdens both socially and economically. Statistics documented that CAD comprised nearly 40% of all death‐relevant causes within developed countries,^1^ and its onset was considered as an interactive outcome of polygenes and environmental parameters.^2^ In spite of numerous studies mentioning the pathogenesis of atherosclerosis and development of ischaemic heart disease, around 30% of CAD cases failed to be explained by known cardiovascular hazards.^3^, ^4^ It was before manifested that CAD could be taken as a consequence of progressive inflammation,^5^ yet the exact mechanisms underlying dysfunctions of CAD remained vague.

Long non‐coding RNAs (lnc RNAs), a type of transcripts comprising >200 nucleotides, were involved in the aetiology of diverse human disorders through epigenetic, transcriptional and post‐transcriptional regulations.^6^, ^7^ Interestingly, it was appeared that lncRNAs also participated in the development of cardiovascular diseases, including heart failure, cardiac hypertrophy, cardiac metabolic diseases and myocardial infarction.^8^ Among them, lncRNA ANRIL, also named as CDKN2BAS, was ranked as the best replicating genetic risk factor for CAD,^8^ and it was speculated to alter expressions of related proteins through RNA interference, gene silencing, chromatin remodelling and DNA methylation.^9^ In addition, the function of lncRNAs was often mediated through the regulation of microRNAs (miRNAs), which post‐transcriptionally regulate gene expression by binding to the 3′ untranslated region (UTR) of mRNAs.^10^ For instance, it was documented that ANRIL modifying miR‐181b could boost a series of vicious transformations that were relevant to human vascular inflammation.^11^ However, whether ANRIL would impact on miR‐181b to regulate the onset or progression of CAD remained unanswered.

The miR‐181 pointed out here has been revealed to affect different aspects of cell life activities, including cell proliferation, cell differentiation and cell death.^12^, ^13^ Furthermore, miR‐181b was discovered to inhibit NF‐κB‐mediated endothelial cell activation by lowering the expression of importin‐α3 (IPOA3), a key protein assisting in translocation of NF‐κB from cytoplasm to nucleus.^14^ Notwithstanding, the role of miRNA‐181b in chronic inflammatory disease, such as atherosclerosis, has not been examined. However, in the vascular endothelium, NF‐κB activation induces the expression of pro‐inflammatory genes, which seemed as a pivotal parameter for the occurrence and development of atherosclerosis.^15^, ^16^, ^17^

All in all, it was hypothesized that ANRIL, miR‐181b and NF‐κB might function to modify the aetiology of atherosclerosis. In response, this study was designed to investigate whether ANRIL regulated the presence and progression of CAD by acting on downstream miR‐181band NF‐κB signalling.

## 2 MATERIALS AND METHODS

### 2.1 Inclusion of subjects

Altogether 327 CAD patients diagnosed by angiography were included from the 455th Hospital of Chinese People's Liberation Army during the time span stretching from March 2015 to July 2016. The participants with concurrent cancer, acute myocardial infarction, severe heart failure (left ventricular ejection fraction 30%), cardiomyopathy, active infection and connective tissue disease were excluded. This study has obtained the informed consents from all patients, and it was approved by the 455th Hospital of Chinese People's Liberation Army and the ethics committee of the 455th Hospital of Chinese People's Liberation Army.

### 2.2 Establishment of mice models and sample collection

The SD rats of clean grade (male; 7‐week; 220‐250 g) were provided by the experimental animal centre of the 455th Hospital of Chinese People's Liberation Army. The rats were anaesthetized with usage of 50 mg/kg pentobarbital sodium. Their four limbs were subcutaneously inserted with needle electrodes, and changes of electrocardiogram were monitored. After tracheal intubation, the rats’ breath was controlled at 90 times/min, and tidal volume was set as 3‐4 mL. The rats were disinfected and operated with thoracotomy before their pericardium was cut. After 30‐minute successful ligation, the artery clamp was loosen, and reperfusion was sustained for 90 minutes to cause ischaemia reperfusion injury. About 10 minutes later, ligation was considered as successful if ST‐segment elevation was observed within electrocardiogram, and those without ST‐segment elevation was sifted out. The cardiac tissues of rats were taken out, and the ischaemic myocardium within left ventricle of the heart was clipped and was frozen within liquid nitrogen at −80°C.

### 2.3 Determination of cardiac function

Sacculus was inserted through atrioventricular valve, and pressure transducer was connected with the computer. Then left ventricular systolic pressure (LVSP), maximum change rate of left intraventricular pressure (±dp/dtmax) and coronary blood‐flow volume (CF) were recorded.

### 2.4 Cell culture

Human coronary endothelial cells (HCAECs) and human umbilical vein endothelial cells (HUVECs) were purchased from the American Type Culture Collection (ATCC). HUVECs and HCAECs were cultured within endothelial cell growth medium (ScienCell, USA) that was supplemented with 5% (v/v) FBS, 1% endothelial cell growth supplement (ECGS) and 1% penicillin streptomycin. The HUVECs and HCAECs within 3‐7 passages were applied for the following experiments.

### 2.5 Conduction of ELISA

The interleukin‐6 (IL‐6) and IL‐8 concentrations were detected exactly in line with the procedures presented within IL‐6 kit (R&D System, Minneapolis, MN, USA) and IL‐8 kit (R&D System, Minneapolis, MN, USA). Finally, after supplementation of stopping solution, the absorbance (A) of each sample was determined at the wavelength of 450 nm by microplate reader (Tecan, Männedorf, Switzerland).

### 2.6 Quantitative real‐time polymerase chain reaction (qRT‐PCR)

RNeasy Mini Kit (Qiagen, Duesseldorf, Germany) was employed to extract total RNA from tissues. The purity and concentration of the RNA were measured with the aid of a spectrophotometer (Thermo Scientific, Waltham, MA, USA). We adopted PrimeScript RT reagent kit (Invitrogen, Carlsbad, CA, USA) to reversely transcribe 1 mg RNA, following the procedures of: (a) at 42°C for 60 minutes, (b) at 95°C for 5 minutes, and (c) at 4°C for 10 minutes. The obtained cDNAs were subject to PCR according to the instructions of the SYBR Green master kit (Applied Biosystems, Foster City, CA, USA). The primers (Table 1) were designed with usage of ABI Primer Express software and were synthesized by Shanghai Sango. Also the specific reaction conditions for ANRIL worked as follows: (a) pre‐denaturation at 95°C for 30 seconds; (b) 40 cycles of denaturation at 95°C for 2 minutes and annealing at 55°C for 1 minute; and (c) extension at 40°C for 5 minutes. The PCR conditions for miR‐181b were particularized as: (a) pre‐denaturation at 95°C for 30 seconds; (b) 40 cycles of denaturation at 95°C for 5 seconds and annealing at 60°C for 30 seconds; and (c) extension at 40°C for 5 minutes. Moreover, the detailed PCR conditions were enlisted as: (a) pre‐denaturation at 95°C for 30 seconds; (b) 40 cycles of denaturation at 95°C for 5 seconds and annealing at 60°C for 30 seconds; and (c) extension at 60°C for 5 minutes. GADPH was designated as the internal reference for ANRIL and NF‐κB, and U6 was set as the internal reference for miR‐181b. The 2^−ΔΔCt^ method^18^ was used to calculate the relative expression of target genes.

**Table 1:** The primer sequence of miR‐181b, lncRNA ANRIL, NF‐κB, TNF‐α, iNOS, ICAM‐1,VCAM‐1 used in this study

Items	Primer sequence
miR‐181b	5′‐ACACTCCAGCTGGGAACATTCATTGCTGTCGG‐3′
	5′‐TGGTGTCGTGGAGTCG‐3′
U6	5′‐CTCGCTTCGGCAGCACA‐3′
	5′‐AACGCTTCACGAATTTGCGT‐3′
LncRNA ANRIL	5′‐TTATGCTTTGCAGCACACTGG‐3′
	5′‐GTTCTGCCACAGCTTTGATCT‐3′
NF‐κB	5′‐AACACTGCCGACCTCAAGAT‐3′
	5′‐CATCGGCTTGAGAAAAGGAG‐3′
TNF‐α	5′‐CTCCAGCTGGAAGACTCCTCCCAG‐3′
	5′‐CCCGACTACGTGCTCCTCACC‐3′
iNOS	5′‐TGGCTTGCCCTTGGAAGTTTCTC‐3′
	5′‐TCCAGGCCATCTTGGTGGCAAGA‐3′
ICAM‐1	5′‐CGACTGGACGAGAGGGATTG‐3′
	5′‐TTATGACTGCGGCTGCTACC‐3′
VCAM‐1	5′‐GCAAGGTTCCTAGCGTGTAC‐3′
	5′‐GGCTCAAGCATGTCATATTCAC‐3′
GAPDH	5′‐ACAGCAACAGGGTGGTGGAC‐3′
	5′‐TTTGAGGGTGCAGCGAACTT‐3′

### 2.7 Cell transfection

The shRNA (shRNA‐ANRIL) targeting ANRIL, the shRNA lentivirus particles added with interfering nucleotides (shRNA‐NC), pcDNA3.1‐ANRIL, miR‐181b mimic, miR‐181b inhibitor and miR‐NC were all gained from Genepharma (Shanghai, China). The pcDNA™3.1 that was resistant to ampicillin was purchased from Invitrogen. The lipopolysaccharides (LPS, *E. coli 0111:B4*) and pyrrolidine dithiocarbamate (PDTC, Sigma, St. Louis, MO, USA) were, respectively, the activator and inhibitor for NF‐κB. The transfection was performed as per the instructions of Lipofectamine™ 2000 (Invitrogen), and cells of each group were collected 24‐48 hours after transfection.

### 2.8 Colony formation assay

The transfected cells were seeded at 1 × 104 within culture dishes of a 35‐mm diameter. The cells stained with trypan blue were observed and counted in triplicate within 6 weeks. Then cells were dissociated and were suspended in the medium containing 0.3% agar. The colonies exceeding 0.5 mm in diameter were counted after 14 days.

### 2.9 Cell proliferation assay

All the procedures were carried through in line with the instructions of CCK‐8 kit (Dojindo Laboratories, Kumamoto, Japan). After 72‐hour transfection, 2 × 10^4^ cells (100 μL) were inoculated within each well of 96‐well culture plate. About 3‐4 hours later when cells grew against the wall, 100 μL RPMI 1640 complete medium and 10 μL CCK‐8 were added. Then, the cells were cultured in 5% CO_2_ at 37°C for 2 hours. The D450 values would be determined with a microplate reader (infinite M200, Tecan, Austria) at the wavelength of 450 nm.

### 2.10 Cell apoptosis assay

When cells were re‐suspended within 500 μL binding buffer, 5 μL Annexin V‐fluorescein isothiocyanate (FITC) (Invitrogen) and 10 μL propidium iodide (PI) were added in the darkness to stain the cells for 15 minutes. Within the scatter diagram drawn from bivariate flow cytometer (Bio‐Rad, Hercules, CA, USA), cells within the lower‐left quadrant and the upper‐left quadrant were, respectively, held as FITC‐/PI‐labelled live cells and FITC‐/PI+ labelled live cells. Moreover, cells within the upper‐right quadrant were considered as necrotic cells labelled with FITC+/PI+, and ones within the lower‐right quadrant were deemed as early apoptotic cell labelled by FITC+/PI‐.

### 2.11 Western blotting

We prepared RIPA lysis buffer to extract proteins from tissues and cells, and Bradford method was employed to measure the protein concentration. Subsequently, equal amounts (ie, 30 μg) of proteins were electrophoresed on the 6% or 10% polyacrylamide gel, and the isolated proteins were then transferred onto the polyvinylidene fluoride (PVDF) membrane. After 1‐hour blockage of the membranes with TBS Tween‐20 buffer that contained 5% bovine serum albumin (BSA) at room temperature, rabbit anti‐human NF‐κB monoclonal antibody (1:900, Abcam, Cambridge, MA, USA), rabbit anti‐human VCAM‐1 monoclonal antibody (1:1000, Abcam Cambridge, MA, USA), rabbit anti‐human VEGF polyclonal antibody (1:20, Santa Cruz Biotechnology, Santa Cruz, CA, USA), rabbit anti‐human HSP70 monoclonal antibody (1:100, Cell Signaling Technology, Danvers, MA, USA), rabbit anti‐human COX‐2 monoclonal antibody (1:100, DAKO, Glostrup, Denmark) and rabbit anti‐human GAPDH monoclonal antibody (1:1000, Cell Signaling Technology, Danvers, MA, USA) were supplemented. Then, the membranes were incubated with corresponding mouse anti‐rabbit secondary antibodies (1:10000, CST) at room temperature for 1 hour, and electro‐chemi‐luminescence (ECL) detection reagent (Millipore, Billerica, MA, USA) was utilized for development. At last, images were collected by employing Syngene Gene Genius gel imaging system (Syngene, Cambridge, UK).

### 2.12 Dual luciferase reporter gene assay

The ANRIL fragments that covered specific miR‐181b binding sites were cloned into the pmirGLO dual luciferase expression vector (Promega, Madison, WI, USA) to construct reporter vectors named as pmirGLO‐ANRIL‐Wt. The same vector including miR‐181b mutational sites within the ANRIL sequence was called as pmirGLO‐ANRIL‐Mut. The HCAECs that have been transfected with miR‐181b and miR‐NC were again transfected with pmirGLO‐ANRIL‐WT or pmirGLO‐ANRIL ‐MUT. Similarly, certain NF‐κB fragments that contained miR‐181b binding sites were amplified by PCR and were then cloned into pmirGLO dual luciferase expression vector to form NF‐κB‐Wt. The NF‐κB‐Mut was produced in a manner same to NF‐κB‐Wt, except that the miR‐181b binding sites within NF‐κB were mutated. When cell confluency reached 40% ‐50%, the cells that have been transfected with miR‐181b mimics or miR‐NC were, respectively, transfected with NF‐κB‐Wt, NF‐κB‐Mut or pRL‐TK reporter vector. Then, a luciferase reporter assay was performed based on the dual‐luciferase reporter gene detection system (Promega).

### 2.13 Statistical analyses

All the statistical analyses were performed with the aid of GraphPad Prism software (GraphPad Prism Software Inc., San Diego, USA). Student's *t* test or one‐way ANOVA test was employed to compare quantitative data (mean ± SD), and chi‐square test was applied for evaluation of enumeration data. A *P* value < 0.05 was considered as a mark of significant differences.

## 3 RESULTS

### 3.1 Association of ANRIL and miR‐181b expressions with baseline characteristics of CAD patients

**Figure 1:**
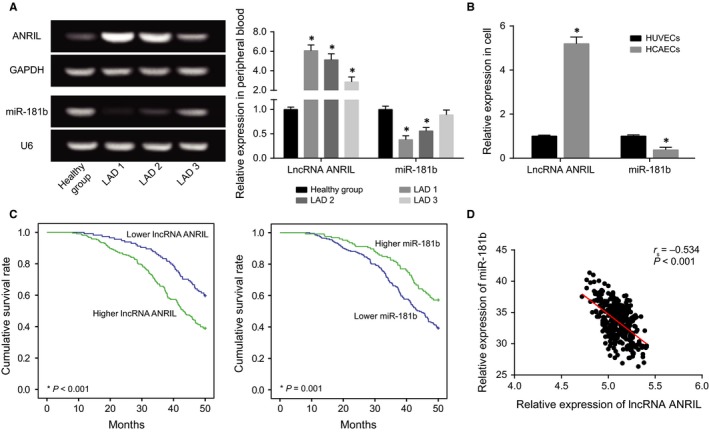
The Expressions of lncRNA ANRIL and miR‐181b were Compared between Coronary Artery Disease (CAD) Tissues/Cells and Normal Tissues/Cells. A, ANRIL and miR‐181b expressions were compared between CAD tissues and normal tissues. *P < 0.05 when compared to normal tissues. B, ANRIL and miR‐181b expressions were compared between HUVECs and HCAECs. *P < 0.05 when compared to HUVECs. C, The survival rates of CAD patients with differentially expressed ANRIL and miR‐181b were compared. D, ANRIL expressions were negatively correlated with miR‐181b expressions within CAD tissues

Founded on the percentage of coronary artery stenosis, the CAD patients were categorized into LAD1 group (81%‐100%, severe), LAD2 group (51%‐80%, moderate), LAD 3 group (30%‐50%, mild) and healthy group. According to Figure 1A, up‐regulated ANRIL expression and down‐regulated miR‐181b expression were observed within CAD patients when compared to the healthy group (*P* < 0.05). It was further revealed that ANRIL expression increased with the rising degree of LAD (*P* < 0.05), yet miR‐181b expression followed a tendency opposite to ANRIL (*P* < 0.05). Similar to the results of tissues, ANRIL was expressed more within HCAECs than within HUVECs, and miR‐181b expression within HCAECs appeared lower than within HUVECs (*P* < 0.05)(Figure 1B). More than that, both highly expressed ANRIL and lowly expressed miR‐181b were associated with CAD population aged over 60 years old, with smoking history, with symptoms of hypertension and hyperlipidemia, with CHOL ≥ 4.34 mmol/L, TG ≥ 1.93 mmol/L and Hcy ≥ 16.8 μmol/L (all *P* < 0.05) (Table 2). Furthermore, the multi‐variate analyses informed us that higher ANRIL expression (HR = 2.10, 95% CI: 1.24‐3.57, *P* = 0.006), lower miR‐181b expression (HR = 1.81, 95% CI: 1.08‐3.02, *P* = 0.024), TG level ≥ 1.93 mmol/L (HR = 3.95, 95% CI: 2.10‐7.46, *P* < 0.001) and Hcy ≥ 16.8 μmol/L (HR = 2.30, 95% CI: 1.30‐4.07, *P* = 0.004) were all correlated with poorer prognosis of CAD patients (Table 3, Figure 1C). At the same time, Spearman's correlation analysis unfolded that miR‐181b expressions were negatively correlated with ANRIL expressions among the CAD patients investigated (rs = −0.534, *P* < 0.001) (Figure 1D).

**Table 2:** Association between the expression of long non‐coding RNA ANRIL/miR‐181b and the clinical characteristics in 327 patients with coronary artery disease

Characteristics	LncRNA ANRIL expression			miR‐181b expression		
	Low	High	*P* value	Low	High	*P* value
Age (mo)						
<60	50	59	**0.003**	55	54	**0.004**
≥60	64	154		146	72	
Gender						
Female	50	72	0.073	70	52	0.241
Male	64	141		131	74	
Distribution						
Single vessel disease	40	63	0.318	63	40	0.989
Double vessel disease	38	65		63	40	
Triple vessel disease	36	85		75	46	
Disease types						
AMI	32	60	0.788	62	30	0.298
UAP	39	80		79	50	
SAP	43	73		60	46	
Family history						
Negative	12	20	0.741	16	16	0.161
Positive	102	193		185	110	
Smoking						
Positive	25	81	**0.003**	75	31	**0.017**
Negative	89	132		126	95	
Hypertension						
Positive	42	110	**0.011**	107	45	**0.002**
Negative	72	103		94	81	
Diabetes						
Positive	32	40	0.053	47	25	0.452
Negative	82	173		154	101	
Hyperlipidemia						
Positive	40	115	**0.001**	105	50	**0.027**
Negative	74	98		96	76	
CHOL (mmol/L)						
≥4.34	56	135	**0.013**	126	65	**0.048**
<4.34	58	78		75	61	
TG (mmol/L)						
≥1.93	60	140	**0.021**	135	65	**0.005**
<1.93	54	73		66	61	
Hcy (μmol/L)						
≥16.8	65	156	**0.003**	146	75	**0.014**
<16.8	49	57		55	51	

AMI, acute myocardial infarction; UAP, unstable angina pectoris; SAP, stable angina pectoris; CHOL, cholesterol; TG, triacylglycerol; Hcy, homocysteine. The bold treatment indicated a significant result featured by *p*<0.05.

**Table 3:** Univariate and multivariate analysis of factors influencing survival in patients with coronary artery disease

Characteristics	Univariate analysis			Multivariate analysis		
	Hazard Ratio	95% CI	*P* value	Hazard Ratio	95% CI	*P* value
LncRNA ANRIL expression						
High vs Low	2.32	1.46‐3.69	**<0.001**	2.1	1.24‐3.57	**0.006**
miR‐181b expression						
Low vs High	2.06	1.31‐3.24	**0.002**	1.81	1.08‐3.02	**0.024**
Age (mo)						
<60 vs ≥60	0.91	0.57‐1.45	0.695	0.99	0.58‐1.68	0.965
Smoking						
Positive vs Negative	1.66	1.04‐2.67	**0.035**	1.23	0.72‐2.10	0.446
Hypertension						
Positive vs Negative	0.91	0.59‐1.41	0.687	0.67	0.40‐1.11	0.118
Hyperlipidemia						
Positive vs Negative	1.69	1.09‐2.62	**0.019**	0.93	0.55‐1.58	0.8
CHOL (mmol/L)						
≥4.34 vs <4.34	1.77	1.13‐2.75	**0.012**	0.56	0.30‐1.06	0.075
TG (mmol/L)						
≥1.93 vs <1.93	3.66	2.29‐5.85	**<0.001**	3.95	2.10‐7.46	**<0.001**
Hcy (μmol/L)						
≥16.8 vs <16.8	3.16	1.95‐5.13	**<0.001**	2.3	1.30‐4.07	**0.004**

CHOL, cholesterol; TG, triacylglycerol; Hcy, homocysteine. The bold treatment indicated a significant result featured by p<0.05.

### 3.2 Variation of cardiac functions within CAD mice models

**Figure 2:**
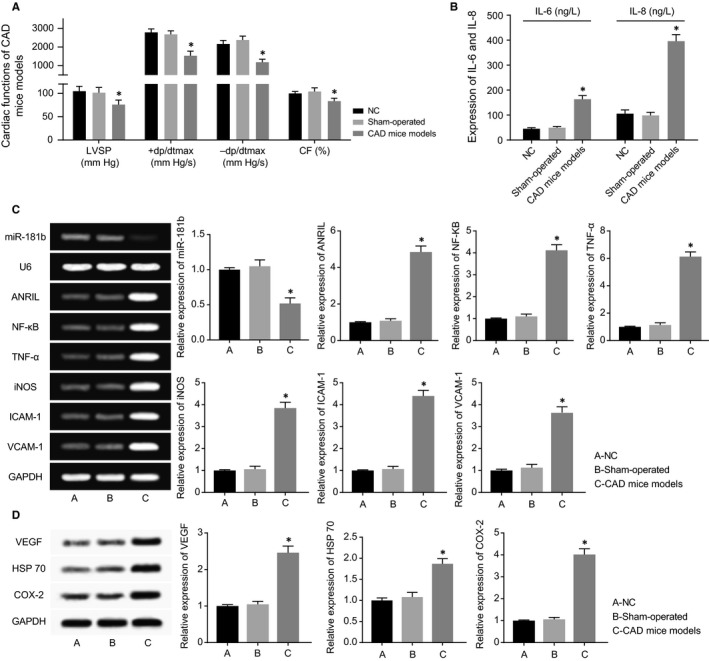
The CAD Mice Models were Established. A, The cardiac functions of mice were compared among CAD mice models, sham‐operated mice and negative control (NC) mice. *P < 0.05 when compared to NC and sham‐operated mice. B, IL‐6 and IL‐8 expressions were compared among CAD mice models, sham‐operated mice and NC mice. *P < 0.05 when compared to NC and sham‐operated mice. C, The expressions of ANRIL, miR‐181b, NF‐κB, TNF‐α, iNOS, ICAM‐1 and VCAM‐1 expressions were compared among CAD mice models, sham‐operated mice and NC mice. *P < 0.05 when compared to NC and sham‐operated mice. D, VEGF, HSP 70 and COX‐2 expressions were compared among CAD mice models, sham‐operated mice and NC mice. *P < 0.05 when compared to NC and sham‐operated mice

When compared to NC group and sham‐operated group, the CAD mice models were observed with remarkably decreased LVSP, +dp/dtmax and ‐dp/dtmax (all *P* < 0.05). Following an analogous trend, CAD mice models exhibited a significantly inhibited CF in comparison with NC group and sham‐operated group (*P* < 0.05) (Figure 2A). However, there was no significant distinction of LVSP, +dp/dtmax, ‐dp/dtmax NC and CF within NC group from those within sham‐operated group (*P* > 0.05).

### 3.3 Expressions of ANRIL, MiR‐181b, NF‐κB, inflammatory factors (IL‐6, IL‐8, TNF‐l, iNOS, ICAM‐1, VCAM‐1 and COX‐2) and vascular‐protective factors (ie, VEGF and HSP 70) within CAD mice models

Up‐regulated ANRIL and NF‐κB expressions, along with largely down‐regulated miR‐181 expressions, were found within AD mice models, with NC and sham‐operated groups as the reference (*P* < 0.05). Besides, regarding the inflammatory factors, IL‐6, IL‐8, NF‐κB, TNF‐, iNOS, ICAM‐1, VCAM‐1 and COX‐2 expressions observed within AD mice models were all beyond those within NC and sham‐operated groups (*P* < 0.05) (Figure 2B,C). Also the vascular‐protective parameters, such as VEGF and HSP 70, were both highly expressed within AD mice models than within mice of NC and sham‐operated groups (*P* < 0.05) (Figure 2D).

### 3.4 ANRIL and miR‐181b regulated survival, viability and apoptosis of HCAECs, along with EMT‐specific proteins within HCAECs

**Figure 3:**
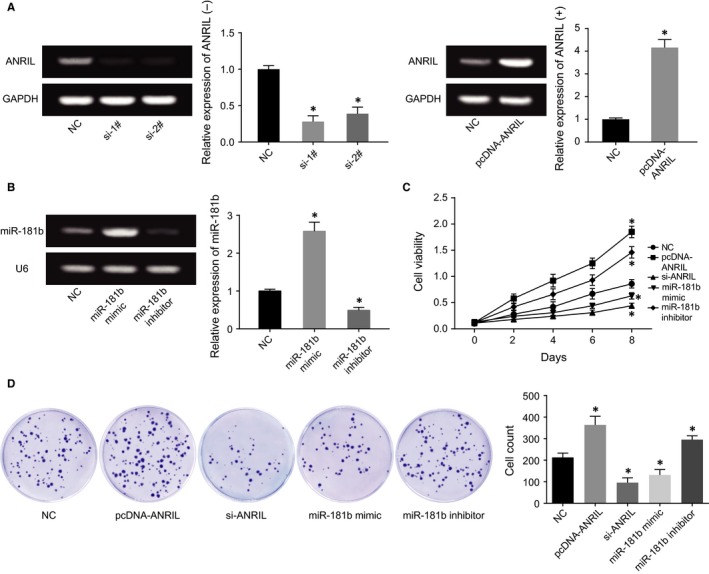
The Effects of ANRIL and miR‐181b on Viability and Survival of HUVECs and HCAECs were Evaluated. A, ANRIL expressions were determined after transfection of si‐1#, si‐2# or pcDNA‐ANRIL. *P < 0.05 when compared to NC. B, MiR‐181b expressions were determined after transfection of miR‐181b mimic or miR‐181b inhibitor. *P < 0.05 when compared to NC. C, The viability of HCAECs was compared among pcDNA‐ANRIL, si‐ANRIL, miR‐181b mimic, miR‐181b inhibitor and NC groups. *P < 0.05 when compared to NC. D, The survival rates of HCAECs were compared after transfection of pcDNA‐ANRIL, si‐ANRIL, miR‐181b mimic, miR‐181b inhibitor or NC. *P < 0.05 when compared to NC

**Figure 4:**
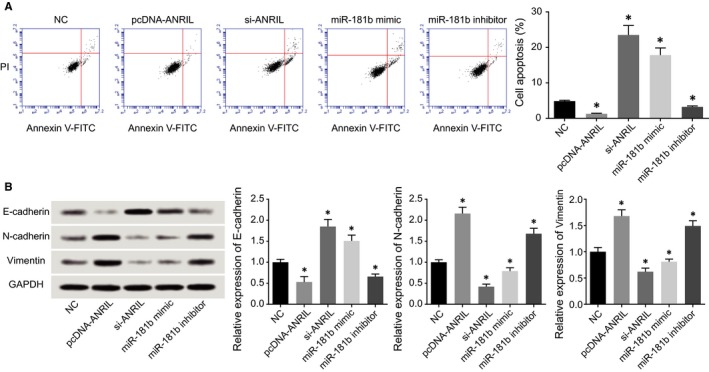
The Influences of ANRIL and miR‐181b on the Apoptosis and Epithelial Mesenchymal Transition (EMT)‐Specific Proteins were Assessed. A, The apoptotic statues of HCAECs were compared after transfection of pcDNA‐ANRIL, si‐ANRIL, miR‐181b mimic, miR‐181b inhibitor and NC groups. *P < 0.05 when compared to NC. B, The expressions of EMT‐specific proteins within HCAECs were compared after transfection of pcDNA‐ANRIL, si‐ANRIL, miR‐181b mimic, miR‐181b inhibitor or NC. *P < 0.05 when compared to NC

ANRIL expressions were up‐regulated and down‐regulated after respective transfection of si‐1#/2# and pcDNA‐ANRIL (*P* < 0.05), and miR‐181b also was over‐expressed and under‐expressed after respective treatments of miR‐181b mimic and miR‐181b inhibitor (Figure 3A,B). It was indicated from CCK8 assay and plate clone formation assay that transfection of either pcDNA‐ANRIL or miR‐181b inhibitor could significantly fortify HCAECs’ viability and put on their survival rate (*P* < 0.05), but HCAECs’ viability and survival rate dramatically fell off within si‐ANRIL and miR‐181b mimic groups in comparison with NC group (*P* < 0.05) (Figure 3C,D). In addition, pcDNA‐ANRIL and miR‐181b inhibitor, which went contrary to si‐ANRIL and miR‐181b mimic, were both correlated with declined apoptotic percentage of HCAECs, when compared to NC group (*P* < 0.05) (Figure 4A). Furthermore, over‐expressed ANRIL and under‐expressed miR‐181 generated lower E‐cadherin expression, along with higher N‐cadherin and vimentin expressions than NC group, which displayed a tendency opposite to under‐expressed ANRIL and over‐expressed miR‐181 (*P* < 0.05) (Figure 4B).

### 3.5 ANRIL and miR‐181b modulated HCAECs’ release of inflammatory factors and vascular‐protective factors

**Figure 5:**
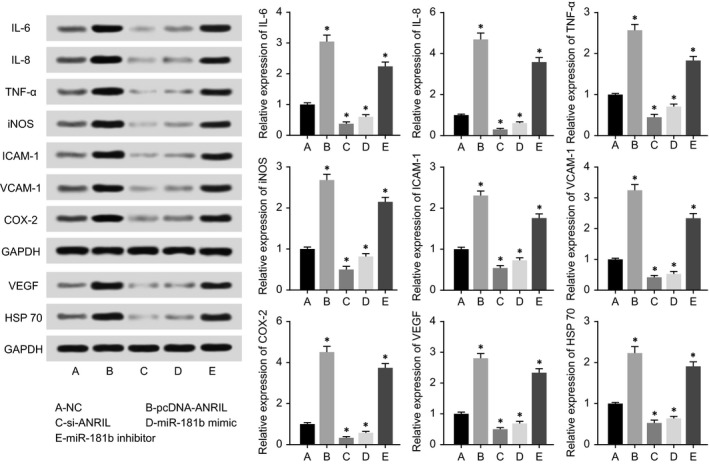
The Expressions of IL‐6, IL‐8, TNF‐α, iNOS, ICAM‐1, VCAM‐1, COX‐2, VEGF and HSP70 within HCAECs were Compared among pcDNA‐ANRIL, si‐ANRIL, miR‐181b Mimic, miR‐181b Inhibitor and NC Groups. *P < 0.05 when compared to NC

In line with Figure 5, we found that HCAECs transfected with pcDNA‐ANRIL and miR‐181b inhibitor released a larger amount of inflammatory factors (ie, IL‐6, IL‐8, TNF‐α, iNOS, ICAM‐1, VCAM‐1 and COX‐2) and vascular‐protective parameters (ie,. VEGF and HSP 70) than NC group. Conversely, silencing of ANRIL or addition of miR‐181b mimic both gave rise to down‐regulated expressions of the inflammatory factors and vascular‐protective parameters mentioned above (*P* < 0.05).

### 3.6 ANRIL targeted miR‐181b to inhibit its expression

**Figure 6:**
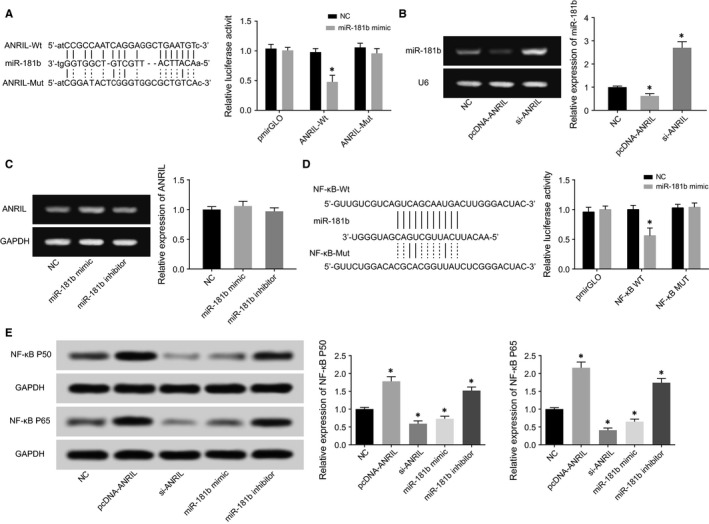
The Correlations among ANRIL, miR‐181b and NF‐kB within HCAECs were Verified. A, The luciferase activities were compared between miR‐181b mimic+ANRIL wt and miR‐181b mimic+ANRIL mut groups. *P < 0.05 when compared to miR‐181b mimic+NC. B, MiR‐181b expressions were detected after transfection of pcDNA‐ANRIL or si‐ANRIL. *P < 0.05 when compared with NC. C, ANRIL expressions were detected after transfection of miR‐181b mimic or miR‐181b inhibitor. D, The luciferase activities were compared between miR‐181b mimic+NF‐kB wt and miR‐181b mimic+NF‐kB mut groups. *P < 0.05 when compared to miR‐181b mimic+NC. E, NF‐kB p50 and p65 expressions were detected after transfection of pcDNA‐ANRIL, si‐ANRIL, miR‐181b mimic or miR‐181b inhibitor. *P < 0.05 when compared to NC

The luciferase activity of the pmirGLO‐ANRIL‐Wt+miR‐181b group seemed quite lower than that of pmirGLO‐ANRIL‐Mut+miR‐181b group (*P* < 0.05), and scarcely any differences were viewed between pmirGLO‐ANRIL‐Mut+miR‐181b group and pmirGLO+NC group (*P* > 0.05) (Figure 6A). In addition, qRT‐PCR results delivered a message that highly expressed ANRIL also notably brought down miR‐181b expression and raised p50/p65 expressions within HCAECs (*P* < 0.05), yet altered miR‐181b expression exerted few effects on ANRIL expression within HCAECs (*P* > 0.05) (Figure 6B,C).

### 3.7 MiR‐181b prohibited HCAECs’ survival, viability and apoptosis, along with EMT‐specific proteins within HCAECs via targeting NF‐κB

The miR‐181b mimic transfected with pmirGLO‐NF‐κB‐Wt displayed a luciferase activity smaller than miR‐181b mimic+pmirGLO‐NF‐κB‐Mut group (*P* < 0.05), whose luciferase activity was approximate to that of miR‐NC+pmirGLO group without statistical significance (*P* > 0.05) (Figure 6D). Meanwhile, we found that up‐regulation or down‐regulation of miR‐181b expression could accordingly alter NF‐κB p50 and p65 expressions (*P* < 0.05) (Figure 6E).

The results of CCK8 assay and colony formation assay both showed that the viability and survival status of HCAECs were more vigorous within miR‐NC+NF‐κB activator group than within miR‐NC group (*P* < 0.05), suggesting that NF‐κB suppressed the miR‐181b‐induced effect of weakening proliferation of HCAECs. Moreover, miR‐NC+NF‐κB activator group presented relatively lower apoptosis than miR‐NC group (*P* < 0.05), implying that NF‐κB played an inhibitory role in the promoting effect of miR‐181b on HCAECs’ apoptosis. What's more, miR‐NC+NF‐κB activator group was determined with lowly expressed E‐cadherin and highly expressed N‐cadherin and vimentin, when compared to miR‐NC group (*P* < 0.05).

### 3.8 MiR‐181b repressed HCAECs’ capacity to release inflammatory factors and vascular‐protective factors through modulation of NF‐κB

MiR‐NC+NF‐κB activator group was found to secrete more inflammatory factors (ie, IL‐6, IL‐8, NF‐κB, TNF‐α, iNOS, ICAM‐1, VCAM‐1 and COX‐2) and vascular‐protective factors (ie, VEGF and HSP 70) than miR‐NC group (*P* < 0.05) (Figure 8). And miR‐NC group remained higher release of the above‐mentioned inflammatory factors and vascular‐protective factors than miR‐181b mimic group (*P* < 0.05).

## 4 DISCUSSION

Coronary artery disease, one principal cause for heart disease‐induced death, has been on the rise in its prevalence because of increased populations with obesity and diabetes.^19^, ^20^, ^21^ Currently, the treatments for CAD mainly incorporated chemotherapy, percutaneous coronary intervention, coronary artery bypass grafting and so on, it still brought about high mortality.^22^ One main reason was believed as that the inaccurate and incomplete diagnostic strategies for CAD resulted in a spike in the rate of missed diagnosis. Therefore, early detection of CAD was of great necessity, and it had high application value to develop bio‐diagnostic markers featuring high sensitivity and convenience.

LncRNAs, a class of non‐coding RNAs with a length greater than 200 nt, can be involved in genetic regulation transcriptionally and post‐transcriptionally, and some of them, including Bvrt, Fendrr, ANRIL, MIAT, MyHeart (Mhrt), functioned essentially in the occurrence of heart diseases, including myocardial infarction, cardiomyopathy, heart failure and atherosclerosis.^23^, ^24^, ^25^, ^26^, ^27^, ^28^ ANRIL was a 3.8‐kb‐long non‐coding RNA transcribed from chromosome 9p21, which was a crucial locus of genetic sensitivity for CAD.^29^, ^30^ Multiple single nucleotide polymorphisms (SNPs) of ANRIL, such as rs6475606, rs10757274 and rs2383206, were reckoned as the pathological factors for CAD, and CAD was observably affected by the aberrant expression of ANRIL.^25^ It was additionally demonstrated that ANRIL could recruit CBX7 of polycomb repressive complex 1 (PRC1) and suz12 of PRC2 within prostate tissues and fibroblasts, so that expressions of INK4b/ARF/INK4a locus would be restrained.^31^, ^32^ The CDKN2A/B therein encoded cell cycle regulatory proteins, such as p16^INK4A^ and p15^INK4B^,^32^ suggesting that ANRIL was actively involved in modifying cell proliferation, motility and apoptosis. Our study also demonstrated that ANRIL could greatly influence the viability, apoptosis and survival percentage of myocardial cells (Figures 3 and 4).

**Figure 7:**
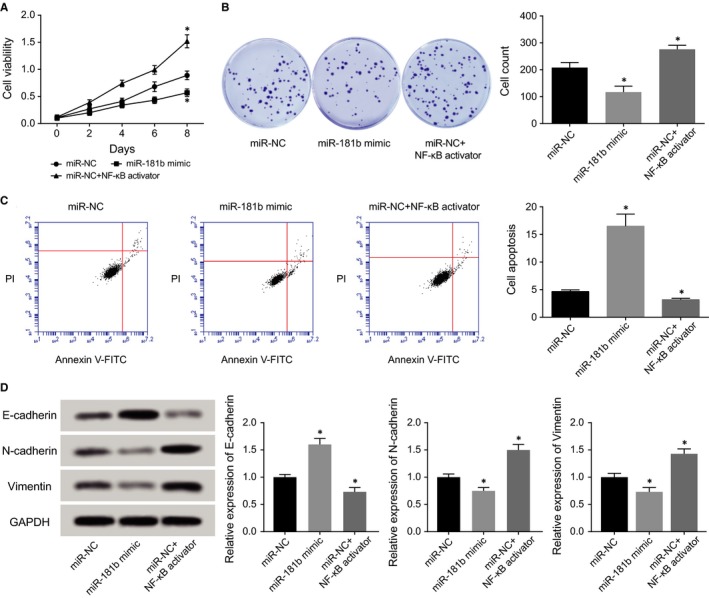
NF‐kB was Subject to Regulation of miR‐181b in Modulating Viability (A), survival (B), apoptosis (C) and epithelial‐mesenchymal transition (EMT)‐specific proteins (D). *P < 0.05 when compared to miR‐NC

**Figure 8:**
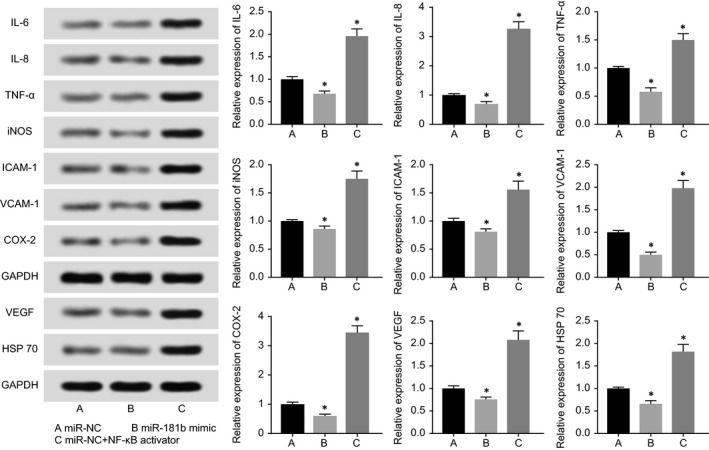
NF‐kB modified by miR‐181b participated in regulating expressions of IL‐6, IL‐8, TNF‐α, iNOS, ICAM‐1, VCAM‐1, COX‐2, VEGF and HSP70 within HCAECs. *P < 0.05 when compared to miR‐NC

More than that, inflammation has been running through the mechanisms that facilitated the onset and development of CAD, especially when coronary plaques were initially formed, unsteadily progressed and finally broken.^33^ For instance, vascular endothelial cells (ECs) could perceive the pathological stimulus within blood by secreting inflammatory factors, and thereby triggering atherosclerosis. Zhou et al^34^ firstly disclosed that ANRIL and related inflammatory factors were up‐regulated within ECs under the stimulation of pathogens, which was mediated by up‐regulated TNF‐α that was caused by NF‐κB signalling. Also ANRIL was capable of binding to PRC‐associated protein (ie, Yin Yang 1) to induce or restrain genetic expressions (eg, IL6 and IL8) that were related with inflammatory responses.^35^, ^36^, ^37^ Thus, it was quite acceptable that ANRIL could act on NF‐κB signalling to induce inflammation, thereby generating CAD‐specific cell activities (Figures 3, 4, 5, 6, 7, 8).

LncRNAs usually modulated disease progression through functioning on coding or non‐coding genes to boost or hinder the regulatory network within cellular processes.^38^, ^39^ This investigation held that the regulatory role of ANRIL in CAD was played via regulation of miR‐181b (Figures 3, 4, 5, 6), which has been a focus among CAD studies. In particular, plasma miR‐181b was under‐expressed remarkably within CAD patients than within normal subjects.^14^ Furthermore, it was mirrored that decreased miR‐181b restrained vascular remodelling by activating TGF‐β/pSmadD2/3 pathway,^40^ and addition of angiotensin II into cardiac fibroblasts could significantly down‐regulate miR‐181b expression.^41^ In addition, it has been illuminated that miR‐181b might suppress breast cancer cells’ capacity of metastasis by targeting CXCL1 and CXCL2, a couple of inflammatory cytokines.^42^, ^43^, ^44^ He et al^45^ arrived at conclusions that miR‐181b was highly expressed with prostate cancer tissues and cell line (ie. PC‐3), and transfecting miR‐181b anti‐sense nucleotide sequences into PC‐3 cell line could result in increased cell apoptosis, decreased cell proliferation and inhibited cell invasion. Also it was covered by Bresin et al^46^ that over‐expressed miR‐181b might lower the expression of anti‐apoptosis proteins (eg, TCL1, Bcl2 and Mcl1) within chronic lymphocytic leukaemia (CLL) cells, and meanwhile, CLL cells’ apoptosis was evidently induced. Besides, miR‐181b expression was markedly up‐regulated within thyroid papillary carcinoma (TPC) tissues, and down‐regulating miR‐181b expression could evidently hinder TPC cells’ proliferation and promoted their apoptosis.^47^ This study also verified that miR‐181b, regulated by ANRIL, participated in the mechanisms underlying proliferation and apoptosis of HCAECs cells (Figures 3, 4, 5, 6).

With respect to inflammatory reactions, it was displayed that MiR‐181b could target Importin‐3(IPOA3) to restrain nuclear transfer of NF‐κB, thereby down‐regulating VCAM‐1 expression and relieving inflammatory responses.^14^ Moreover, after intravenous injection of miR‐181b mimic into atherosclerosis mice, the convergence of macrophages, CD^4+^ T cells and other inflammatory cells were held up, along with decreased expressions of inflammatory markers.^11^ Similar to this, our study verified that miR‐181b could induce inflammatory reactions both in vivo and in vitro, which was specifically manifested as that the expressions of IL‐6, IL‐8, TNF‐α, iNOS, ICAM‐1, VCAM‐1, COX‐2 were down‐regulated (Figures 5 and 8).

The NF‐κB signalling mentioned above not only resulted in incremental apoptosis and declined migration of cancer cells,^48^ but also mainly involved in inflammatory response. NF‐κB could directly stimulate expressions of such inflammatory factors as IL‐6, IL‐8, TNF‐α, iNOS, ICAM‐1, VCAM‐1 and COX‐2 (Figure 8). Among them, increased release of TNF‐α also could induce aggregation and expression of iNOS, ICAM‐1 and VCAM‐1 within the ischaemic region. Also IL‐6 could up‐regulate ICAM‐1 expression within endothelial cells, and thereby aggravating ischaemia to induce myocardial damage.^49^ Another channel of NF‐κB signalling lied in that activation of NF‐κB would trigger myocardial tissues’ awareness of self‐protection, so that enhancive expression of vascular‐protective factors (eg, VEGF and HSP90) could enhance proliferation of epithelium mucosae, tissue recovery and protection of cardiomyocytes (Figure 8).

Above all, this investigation elucidated that ANRIL could target miR‐181b to control proliferation and apoptosis of CAD cells, thereby inducing activation of NF‐κB signalling and release of inflammatory biomarkers, including IL‐6, IL‐8, NF‐κB, TNF‐α, iNOS, ICAM‐1, VCAM‐1 and COX‐2. Nevertheless, this study merely carried out in vitro experiments about the ANRIL/miR‐181b/NF‐κB axis, yet mice CAD models were not established to verify relevant results. Secondly, few experiments were carried out to explore whether overexpression or under‐expression of ANRIL and miR‐181b would affect the phenotypes of mice. Thirdly, we were not informed about if there existed small‐molecule drugs that could interfere with CAD treatment by impeding or promoting ANRIL/miR‐181b/NF‐κB axis. Lastly, curves were not fitted to assess if ANRIL and miR‐181b could act as biomarkers for early‐diagnosis of CAD.

## CONFLICT OF INTEREST

None.
